# Creating a sustainable brain health navigator model to improve the diagnostic journey for Alzheimer's disease: the experience of one health care system in Kentucky

**DOI:** 10.1002/alz70860_101274

**Published:** 2025-12-23

**Authors:** Gregory Cooper, Steven Patton, Deborah Lockridge, Stephanie Freeman

**Affiliations:** ^1^ Norton Neuroscience Institute, Louisville, KY, USA; ^2^ Norton Community Medical Associates, Louisville, KY, USA

## Abstract

**Background:**

The Norton Healthcare (NHC) not‐for‐profit system includes five adult hospitals and one children's hospital in Louisville, KY and three hospitals in southern Indiana. We are the largest healthcare system in the region with around than 22,000 employees and over 2,100 employed medical providers. Norton provides care at more than 400 locations including primary, pediatric, and specialty care practices. Though we have a strong memory disorder clinic and one of the busiest anti‐amyloid programs in the country, our clinic sees a relatively small fraction of the total number of NHC patients who are likely to have mild cognitive impairment (MCI) or dementia. Participating in the US BHN Program will enable us to develop better protocols for detecting patients at risk and those with mild memory impairment as early as possible and to efficiently and effectively navigate them from primary to specialty care for evaluation and, ultimately, treatment.

**Method:**

The proposed pilot site of care is the Norton Community Medical Associates—Preston primary care practice, one of 40 primary care clinics in our system. NCMAPreston is located on the borders of Metro City Council Districts 13 and 24. District 13 has a higher proportion of people living in poverty and low‐income households than the city as a whole. It is critically important to test the DAC‐SP BHN Model in a diverse setting with lower social determinants of health. NHC community medical directors offer direct community outreach and also participate in neighborhood educational health programs that address health and racial inequalities in underserved communities.

**Result:**

We anticipate that group development of the brain health navigator role and processes will yield a more thoroughly considered protocol that is adaptable to the diverse practices across our system. Better navigation will also enable improved coordination of care and utilization of our NNI Resource Center to connect families with needed resources.

**Conclusion:**

The Brain Health Navigator Model and other resources of DAC‐SP support the implementation of boundary spanning roles across primary and specialty care, designed for a sustainable and scalable timely and accurate diagnostic journey across Norton Healthcare and similar health systems.

